# Inhibitory Effects of *Helianthus tuberosus* Ethanol Extract on *Dermatophagoides farina* body-induced Atopic Dermatitis Mouse Model and Human Keratinocytes

**DOI:** 10.3390/nu10111657

**Published:** 2018-11-03

**Authors:** Yun-Mi Kang, Kyou-Young Lee, Hyo-Jin An

**Affiliations:** 1 Department of Pharmacology, College of Korean Medicine, Sangji University, 83 Sangjidae-gil, Wonju-si, Gangwon-do 26339, Korea; yunmi6115@naver.com; 2 Department of Korean Ophthalmology and Otolaryngology and Dermatology, College of Korean Medicine, Sangji University, Wonju, Gangwon-do 26339, Korea; lky0706@naver.com

**Keywords:** atopic dermatitis, *Helianthus tuberosus*, filaggrin, adhesion molecules, NF-κB, MAPK

## Abstract

Atopic dermatitis (AD) is a chronic inflammatory skin disease characterized by complex symptoms. To treat AD without adverse effects, alternative therapeutic agents are required. The tubers of *Helianthus tuberosus* L. (Jerusalem artichoke) have been used in folk remedies for diabetes and rheumatism. However, its effect on AD development remains unknown. Therefore, this study examined the inhibitory effect of *H. tuberosus* (HT) on AD skin symptoms using an NC/Nga mouse model and HaCaT keratinocytes. The effect of HT and associated molecular mechanisms were evaluated in *Dermatophagoides farina* body (Dfb)-induced AD mice and tumor necrosis factor (TNF)-α/interferon (IFN)-γ-stimulated HaCaT keratinocytes by ELISA, western blot, and histological analysis. Topical HT administration attenuated AD skin symptoms in Dfb-induced AD mice, with a significant reduction in the dermatitis score and production of inflammatory mediators. HT also decreased epidermal thickness and mast cell infiltration. Moreover, HT restored filaggrin expression and inhibited adhesion molecules in the mice. These effects were confirmed in vitro. Furthermore, HT suppressed the activation of NF-κB, Akt, and mitogen-activated protein kinase (MAPK) signaling pathways induced by TNF-α/IFN-γ. These results suggest that HT is a potential therapeutic agent or supplement for skin allergic inflammatory diseases such as AD.

## 1. Introduction

Atopic dermatitis (AD) is a chronic allergic inflammatory skin disease characterized by dry, red, cracked, and inflamed skin, erythema, and itching. AD is a disease arising from the complex interaction of genetic, environmental, and psychological factors causing skin barrier dysfunction [1]. *Dermatophagoides farina* body (Dfb) causes the pathogenesis of AD via the induction of immune responses in epidermal keratinocytes [2]. In a previous study, repeated application of Dfb produced AD skin symptoms [3].

Skin barrier dysfunction caused by alteration of skin barrier proteins is one of the main initial factors in the pathogenesis of AD. Filaggrin (a filament aggregation protein) plays a critical role in the differentiation of skin keratinocytes in the stratum granulosum [4]. Decreased expression of filaggrin in the skin and loss-of-function mutations in the filaggrin gene (*FLG*), have been described in AD. Mutations in *FLG* can lead to downstream immunologic activation, leading to the synthesis and secretion of specific immunoglobulin E (IgE) antibodies against allergens, causing abnormalities in the skin barrier [5].

Adhesion molecules, such as intercellular adhesion molecule 1 (ICAM-1), vascular cell adhesion molecule 1 (VCAM-1), and E-selectin are membrane-bound molecules that mediate the attachment of leukocytes to endothelial cells and the control of their retention and migration through the skin [6]. It is reported that the expression of adhesion molecules is up regulated in the skin of patients with AD [7].

*Helianthus tuberosus* L. (Asteraceae family) is a perennial herb originating in eastern North America. Its tuber, which is used for the treatment of diabetes as a source of inulin, contains considerable amounts of fructans, dietary soluble fiber, sesquiterpenes, diterpenes, and chlorogenic acid analogs [8]. *H. tuberosus* exerts aperient, diuretic [9], spermatogenic [10], antipyretic, analgesic, anti-inflammatory, anti-oxidant, and anti-spasmodic effects [11]. It has also previously been reported to act on the skin [12]. Therefore, we hypothesized that *H. tuberosus* may have anti-inflammatory and beneficial effects on AD, which have not previously been investigated. In the present study, we examined whether *H. tuberosus* 30% ethanol extract (HT) alleviated AD skin symptoms in a Dfb-induced mouse model and TNF-α/IFN-γ-stimulated human HaCaT keratinocytes.

## 2. Materials and Methods

### 2.1. Chemicals and Reagents

3-(4,5-Dimethylthiazol-2-yl)-2,5-diphenyl tetrazolium bromide (MTT), dimethyl sulfoxide (DMSO), and all other chemicals were purchased from Millipore Sigma (Billerica, MA, USA). Recombinant human TNF-α and recombinant human IFN-γ were purchased from Bio-Techne Ltd. (Abingdon, UK). Dulbecco’s modified Eagle’s medium (DMEM), fetal bovine serum (FBS), penicillin, and streptomycin were obtained from Life Technologies Inc. (Grand Island, NY, USA). Primary antibodies against p-IKK α/β (cat no. 2697), NF-κB p65 (cat no. 8242), p-Akt (cat no. 9271), and ICAM-1 (cat no. 4915) were obtained from Cell Signaling Technology, Inc. (Danvers, MA, USA). Primary antibodies against IKK α/β (cat no. 7607), p-IκB-α (cat no. 8404), IκB-α (cat no. 203), Akt1/2/3 (cat no. sc-8312), PARP (cat no. sc-9542), α-tubulin (cat no. sc-8035), Filaggrin (cat no. sc-66192), VCAM-1 (cat no. sc-1504), E-selectin (cat no. sc-5262), and β-actin (cat no. sc-81178) were purchased from Santa Cruz Biotechnology Inc. (Dallas, TX, USA). Horseradish peroxidase-conjugated secondary antibodies were purchased from Jackson ImmunoResearch laboratories, Inc. (West Grove, PA, USA). The histamine ELISA kit was obtained from Enzo life Sciences, Inc. (Farmingdale, NY, USA). The ELISA kits for TNF-α and IL-6 were obtained from R&D Systems, Inc. (Minneapolis, MN, USA).

### 2.2. Sample Preparation

Dried tubers of *Helianthus tuberosus* were purchased from Yangwonfood (Cheonan, Korea) and extracted with 30% ethanol (DAEJUNG chemicals & metals, Siheung, Korea). The extract was concentrated under reduced pressure. The decoction was filtered, lyophilized, and stored at 4 °C. The yield of the dried extract from the starting crude materials was 17.33%. To prepare the sample for the in vitro experiment, the extract powder that resulted from the drying process was dissolved in distilled water.

### 2.3. Dfb-Induced AD Model

**Figure 1:**
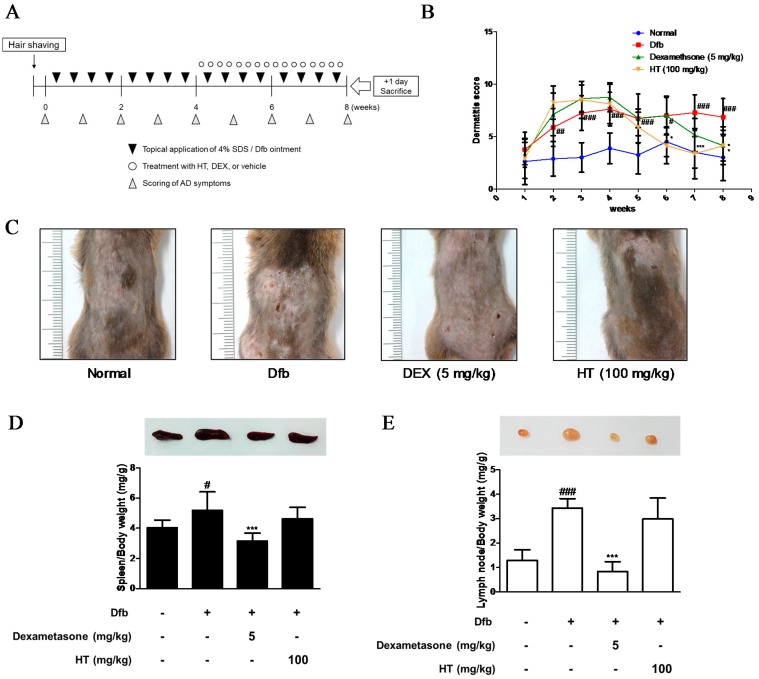
Effects of HT on the development of Dfb-induced AD-skin symptoms in NC/Nga mice. (**A**) Schematic representation of the experiment. Dexamethasone (5 mg/kg, DEX) was used as a positive control. (**B**) Dermatitis scores were measured once a week for 8 weeks. The dermatitis score was defined as the sum of scores graded for each of the symptoms. (**C**) Clinical features of AD-skin symptoms. (**D**–**E**) Representative size and weight of spleen and lymph node were compared by photographic images. Organs/whole body weight were measured. The data shown represent mean ± standard deviation (S.D.) of three independent experiments. ^#^ *p* < 0.05, ^##^ *p* < 0.01, ^###^ *p* < 0.001 vs. the control group; * *p* < 0.05, and *** *p* < 0.001 vs. Dfb-treated group. SDS: sodium dodecyl sulfate; Dfb: *Dermatophagoides farina* body; HT: *H. tuberosus*; AD: Atopic dermatitis.

A total of 32 NC/Nga male mice (6 weeks old; 20–25 g body weight) were obtained from Daehan BioLink (Eumsung, Korea), a branch of Charles River Japan (Kanagawa, Japan) and maintained under constant conditions at a temperature of 20–25 °C, humidity of 40–60%, and a 12 h light/dark cycle. The mice were randomly assigned to one of four groups (*n* = 8 per group): baseline-applied normal group, Dfb-induced group, dexamethasone (Dex; positive control) oral administration group, and HT (100 mg/kg)-treated group. To induce AD-like skin lesions, the shaved dorsal area was topically treated with of 100 mg crude extract *of* Dfb (Biostir-AD; Biostir, Hyogo, Japan). Mite antigen application was repeated twice a week for 8 weeks. Barrier disruption was achieved by 150 μL of 4% sodium dodecyl sulfate (SDS) treatment, 3 h before the application of Dfb ointment. The same volume of baseline (vehicle) was applied to the normal group. After the first challenge inducing the AD-like symptoms for 4 weeks, the mice were topically applied vehicle or HT (100 mg/kg admixed in baseline) and were treated orally with dexamethasone (5 mg/kg dissolved in PBS) 4 h after Dfb treatment once a day. Mice were sacrificed at the end of the experiment. The experimental scheme is summarized in Figure 1A. Skin tissues from the back of the mice were obtained and subjected to histological and western blot analysis. All procedures were performed in accordance with university guidelines and approved by the Ethical Committee for Animal Care and the Use of Laboratory Animals, Korean Medicine, Sangji University (Wonju, Korea; approval no. 2017-15).

### 2.4. Scoring of Dermatitis Severity

Clinical dermatitis severity was evaluated using the method according to the scoring system [13] as follows: The severity of dermatitis was evaluated from the start to the end of the experiment. The development of skin symptoms, erythema/hemorrhage, scarring/dryness, edema, and excoriation/erosion, was scored as follows: 0, none; 1, mild (<20%); 2, moderate (20–60%); or 3, severe (>60%). The individual scores were summed to give the dermatitis score.

### 2.5. Cytokine Assays

Dorsal protein extracts were suspended in PRO-PREP^™^ protein extraction solution (Intron Biotechnology Inc., Seoul, Korea) and incubated for 20 min at 4 °C. Debris was removed via micro-centrifugation at 11,000× *g* for 30 min at 4 °C, followed by rapid freezing of the supernatant. The protein concentration was determined using Bio-Rad protein assay reagent (Bio-Rad Laboratories Inc., Hercules, CA, USA) according to the manufacturer’s protocol. The levels of TNF-α, and IL-6 were quantified using ELISA kits according to the manufacturer’s protocol.

### 2.6. Histamine and IgE Assay

Blood was collected from each mouse at the end of the experiment. Serum was obtained by centrifugation at 1700× *g* for 30 min and stored at −80 °C until analysis. The release of histamine and IgE was measured using an ELISA kit in accordance with the manufacturer’s protocol.

### 2.7. Histopathological and Immunohistochemical (IHC) Analysis

At the end of the study period, the dorsal skin of mice was obtained. The samples were fixed in 10% buffered formalin, embedded in paraffin, sectioned (4 μm thick), and stained with hematoxylin and eosin (H&E) and toluidine blue to detect epidermal thickness and inflammatory cell infiltration, respectively. For immunohistochemical staining, a portion of the skin samples from the back for each group was fixed in 10% formalin. After paraffin embedding, sections were cut and the slides were deparaffinized by xylene, rehydrated in ethanol, and rehydrated with water. Endogenous peroxidase activity was blocked using 0.6% H_2_O_2_ in 50% MeOH and the slides were then treated with 0.3% triton in PBS for permeabilization and pre-blocked with 10% normal goat serum for 1 h, followed by overnight incubation with a specific antibody at 4 °C. After this, sections were washed and incubated with horseradish peroxidase-conjugated secondary antibodies for 1 h at room temperature. The activity was visualized with a 3,3′-diaminobenzidine (DAB) chromogen and counterstained with H&E. Pathological changes of all stained skin sections were observed using a DM IL LED microscope (Leica, Wetzlar, Germany) and photographed using a DFC295 (Leica). Digital images were taken from each slide and analyzed using Leica Application Suite (Leica).

### 2.8. Cell Culture and Sample Treatment

HaCaT keratinocytes were provided by Professor Jae-Young Um (Kyung Hee University, Seoul, Korea) and were grown at 37 °C in DMEM supplemented with 10% FBS, penicillin (100 U/mL), and streptomycin (100 μg/mL) in a humidified atmosphere of 5% CO_2_. HaCaT keratinocytes were seeded at a density of 1 × 10^5^ cell per well, starved with 0.1% FBS media for 24 h, and treated with HT at 100, 200, or 400 μg/mL for 1 h at 37 °C in humidified air with 5% CO_2_ and then stimulated with 10 ng/mL of TNF-α/IFN-γ at 37 °C for the indicated time.

### 2.9. Cell Viability Assay

Cells were seeded in a 96-well culture plate at 5 × 10^4^ cells per well in culture medium and allowed to attach for 24 h. Cells were treated with medium containing various concentrations of HT. After incubating for 24 h, the cells were treated with 50 μL of MTT (5 mg/mL) for 4 h. The formazan precipitated was dissolved in DMSO, and absorbance was measured at 540 nm using a microplate reader.

### 2.10. Western Blot Analysis

Lysates of cells, or dorsal skin tissue were suspended in PRO-PREP^™^ protein extraction solution (Intron Biotechnology, Inc.; Seoul, Korea) and incubated for 20 min at 4 °C. Cell debris was removed via micro-centrifugation at 11,000× *g* for 30 min at 4 °C, followed by rapid freezing of the supernatant. The protein concentration was determined using Bio-Rad protein assay reagent (Bio-Rad Laboratories, Inc.) according to the manufacturer’s protocol. Cellular proteins from the treated and untreated cell extracts were electroblotted onto a polyvinylidene fluoride membrane following separation via 8–12% SDS-PAGE. The membrane was incubated for 1 h with blocking solution (5% skim milk) at room temperature, followed by overnight incubation with the primary antibodies (1:1000) at 4 °C. The blots were washed three times with Tween 20/Tris-buffered saline (T/TBS) and incubated with horseradish peroxidase-conjugated secondary antibody (1:2000) for 2 h at room temperature. The blots were washed three times with T/TBS and then developed via enhanced chemiluminescence (GE Healthcare Life Sciences, Chalfont, UK). Densitometric analysis was performed using Bio-Rad Quantity One software version 4.3.0 (Bio-Rad Laboratories, Inc.).

### 2.11. Statistical Analysis

The data are expressed as the mean ± standard deviation of triplicate experiments. Statistically significant differences were compared using one-way analysis of variance and Dunnett’s post hoc test. *p* < 0.05 was considered to indicate a statistically significant difference. Statistical analysis was performed using SPSS statistical analysis software (version 19.0, IBM SPSS, Armonk, NY, USA).

## 3. Results

### 3.1. HT Attenuated Clinical Severity of AD Skin Symptoms in Dfb-Induced AD Mice

We evaluated the anti-AD effects of HT on NC/Nga mice as an AD mouse model. The dermatitis scores of the dorsal skin in the Dfb-induced groups increased markedly until 8 weeks after induction while those in the control group did not. However, the severity was noticeably attenuated by HT treatment from 5 weeks; the effect of HT was greater than that of dexamethasone, which is widely used to treat AD (Figure 1B,C). Since AD often develops as a systemic immune response, it can affect the immune organs [14]. The spleen and local lymph node should also be examined to quantify atopic symptoms in the mouse model [15]. Therefore, we evaluated morphological changes in the spleen and lymph node. Dfb induced spleen enlargement in mice, but the spleens were reduced to almost normal size in mice treated with HT. We observed an increase in lymph node weight in Dfb-induced mice that was reduced by application of HT, although this result was not significant (Figure 1D,E). These results showed that HT has an alleviatory effect on clinical AD symptoms in Dfb-induced AD mice.

### 3.2. HT Inhibited the Production of Inflammatory Mediators in Dfb-Induced AD Mice

**Figure 2:**
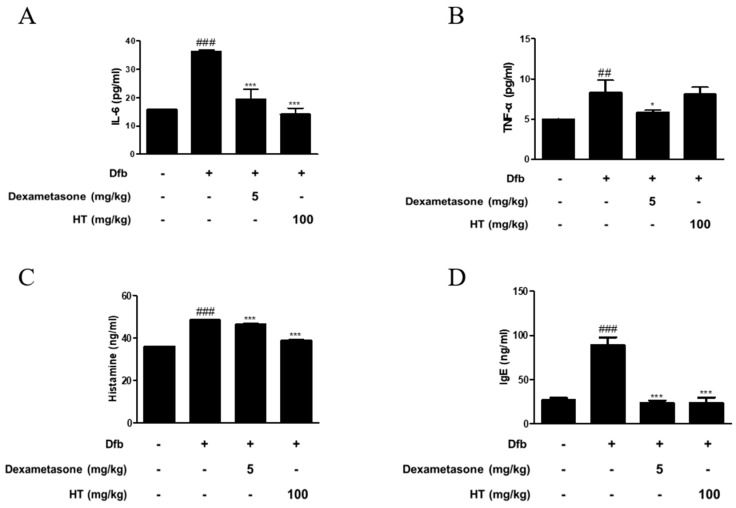
Effects of HT on the production of inflammatory mediators in Dfb-induced NC/Nga mice. The production of (**A**,**B**) pro-inflammatory cytokines, (**C**) histamine release, and (**D**) serum IgE were measured by ELISA. Dexamethasone (5 mg/kg) was used as a positive control. The data shown represent mean ± S.D. of three independent experiments. ^##^ *p* < 0.01, ^###^ *p* < 0.001 vs. the control group; * *p* < 0.05, and *** *p* < 0.001 vs. Dfb-treated group.

AD is a skin disease associated with increased levels of inflammatory cytokines, histamine, and IgE [16]. To confirm the anti-inflammatory effect of HT in Dfb-induced AD mice, we measured inflammatory mediators using ELISA. As shown in Figure 2, Dfb-induced IL-6 levels in the dorsal skin of AD mice were higher by 2-fold than those in the dorsal skin of control mice. This increase was significantly suppressed by treatment with HT. Dfb caused TNF-α levels in the dorsal skin of AD mice (8.29 ± 1.54 pg/mL), but HT slightly decreased the level (8.10 ± 0.84 pg/mL), although it was not significant. Since elevated serum histamine and IgE levels are major characteristics of AD symptoms, their levels in the serum were examined. HT also suppressed the serum levels of histamine and total IgE, compared with the Dfb-induced group (Figure 2C,D). Thus, these results indicate that HT has an anti-AD effect via prevention the release of AD-related inflammatory mediators in Dfb-induced AD mice.

### 3.3. HT Reduced Epidermal Thickness and Mast Cell Infiltration in Dfb-Induced AD Mice Skin

**Figure 3:**
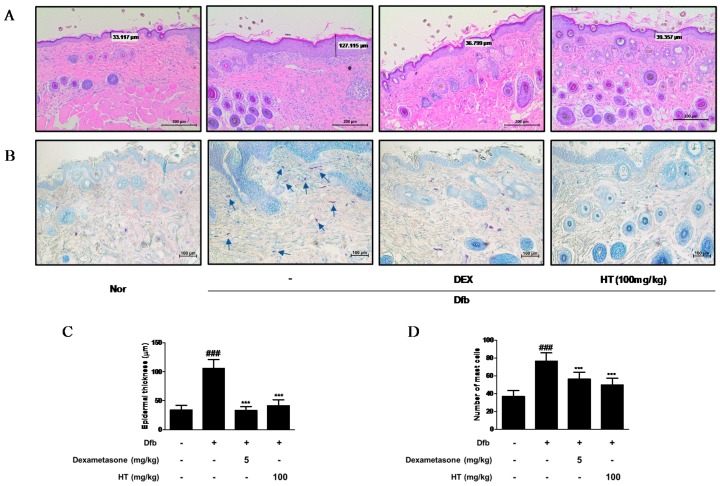
Effects of HT on the histological alterations in Dfb-induced NC/Nga mice. Histological features of the dorsal of NC/Nga mice. Tissues were excised, fixed in 10% formaldehyde, embedded in paraffin, and sectioned. Dexamethasone (5 mg/kg, DEX) was used as a positive control. (**A**) The sections were stained with H&E (scale bar = 200 μm). (**B**) The sections were stained with toluidine blue to identify mast cells. Blue arrows indicated stained mast cells (scale bar = 100 μm). (**C**) Epidermal thickness in H&E stained sections were measured under a microscope. (**D**) Mast cells were counted in toluidine stained sections with a microscope. The data shown represent mean ± S.D. of three independent experiments. ^###^ *p* < 0.001 vs. the control group; *** *p* < 0.001 vs. Dfb-treated group. Nor: normal; H&E: hematoxylin and eosin.

Increased epidermal thickness was due to pathologically activated epidermal proliferation caused by altered differentiation of keratinocytes at the inflamed skin lesions [17]. To histologically evaluate the effect of HT on the skin of AD mice, we performed skin histological analysis using H&E and toluidine blue staining. In H&E-stained skin tissue sections, the Dfb group displayed hyperkeratosis and hypertrophy. The epidermal thickness was greater in the Dfb group than in the normal group, but treatment of HT substantially reduced the thickness of epidermal layer (Figure 3A,C). In the toluidine blue-stained skin tissue sections, mast cell infiltration, a marker of inflammation, was considerably increased in the Dfb group compared to that in the normal group, whereas HT treatment reduced the number of mast cells (Figure 3B,D). These results suggest that topically administered HT may have a potential to improve the histological features of Dfb-induced AD mice skin.

### 3.4. HT Restored Filaggrin Level and Inhibited the Expression of Adhesion Molecules in Dfb-Induced AD Mice Skin

**Figure 4:**
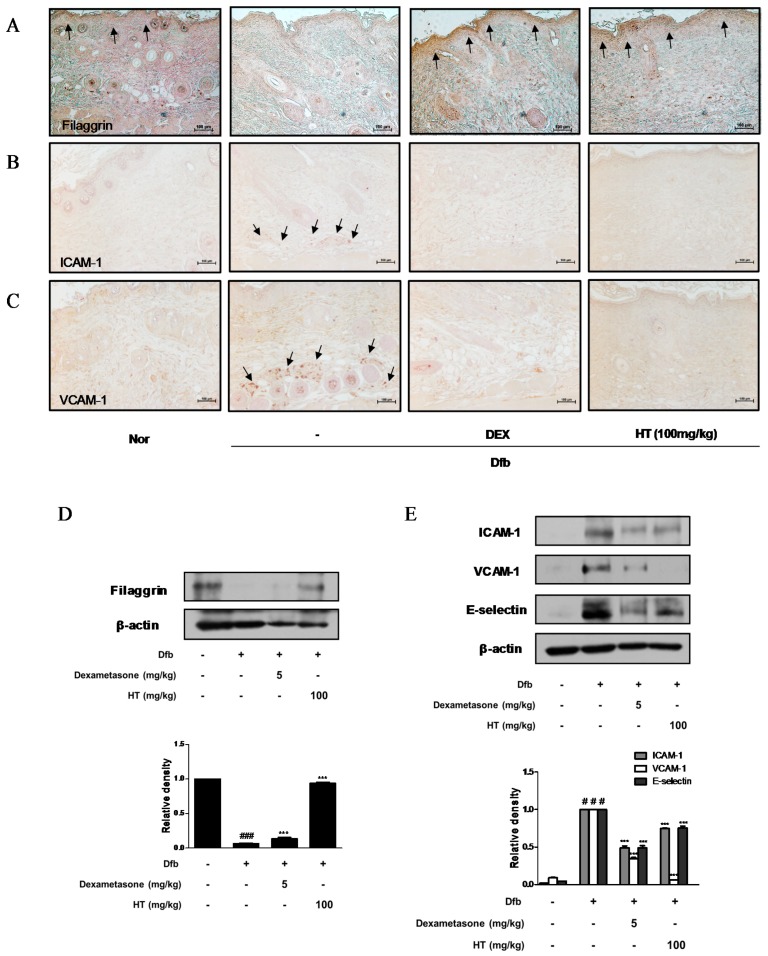
Effects of HT on expression of adhesion molecules and filaggrin in Dfb-induced NC/Nga mice. Skin sections were stained with immunohistochemical staining. Dexamethasone (5 mg/kg, DEX) was used as a positive control. (**A**) Filaggrin, (**B**) ICAM-1, and (**C**) VCAM-1 in the mouse skin were detected using specific antibodies. Black arrows indicated stained (scale bar = 100 μm). Total proteins were prepared and western blotted for (**D**) filaggrin, and (**E**) adhesion molecules using specific antibodies. β-actin was used as internal control. The data shown represent mean ± S.D. of three independent experiments. ^#^ *p* < 0.05, ^###^ *p* < 0.001 vs. the control group; *** *p* < 0.001 vs. Dfb-treated group.

Down regulation of skin barrier proteins such as filaggrin, a differentiation marker of keratinocytes, increases allergen and pathogen penetration and reduces skin hydration [18]. Since alteration of skin barrier proteins is one of the main factors in the pathogenesis of AD, we examined whether HT affects filaggrin expression by IHC. Dfb decreased the expression of filaggrin in the epidermis compared to the control, whereas HT treatment restored the decreased expression of filaggrin to a greater extent than dexamethasone treatment (Figure 4A). We also explored the effects of HT on adhesion molecules which play a key role in the recruitment of immunocytes to inflamed skin and are likely to contribute to AD [19]. Substantial increases in ICAM-1 and VCAM-1 expression were observed in the skin of Dfb-induced AD mice by IHC, indicating that HT treatment significantly decreased ICAM-1 and VCAM-1 protein expression in the dermis of AD mice (Figure 4B,C). These regulatory effects of HT on filaggrin and adhesion molecules were confirmed by western blot analysis. As shown in Figure 4D,E, levels of filaggrin significantly increased in the HT group compared to those in the Dfb group. Elevated ICAM-1, VCAM-1, and E-selectin levels in Dfb-induced mice were decreased markedly by the treatment with HT. These results that HT has an anti-AD effect by protecting skin barrier functions and inhibiting Dfb-induced adhesion molecules in Dfb-induced AD mice.

### 3.5. HT Regulated the Expression of Filaggrin and VCAM-1 in TNF-α/IFN-γ-Stimulated HaCaT Keratinocytes

**Figure 5:**
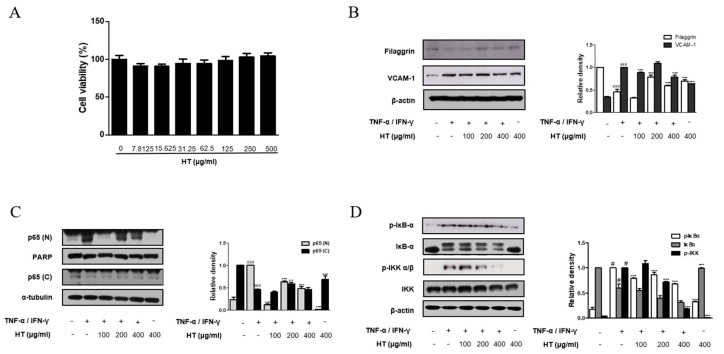
Effect of HT on the expression of filaggrin, VCAM-1 and NF-κB activation in TNF-α/IFN-γ-stimulated HaCaT keratinocytes. (**A**) HaCaT keratinocytes were incubated with the indicated doses of HT for 24 h, and then cell viability was evaluated by MTT assay. HaCaT keratinocytes were pre-treated with HT for 1 h and then stimulated with TNF-α/IFN-γ (10 ng/mL) for 24 h. Total proteins were prepared and western blotted for (**B**) filaggrin and VCAM-1 using specific antibodies. β-actin was used as internal control. (**C**) Cells were pre-treated with HT for 1 h and then stimulated with TNF-α/IFN-γ (10 ng/mL) for 15, 30 min. Nuclear (N) and cytosol (C) extracts were isolated, and levels of p65 in fractions were determined by western blotting. PARP and α-tubulin were used as internal controls. HaCaT keratinocytes were pre-treated with HT for 1 h and then stimulated with TNF-α/IFN-γ (10 ng/mL) for 5-10 min. Total proteins were prepared and western blotted for (**D**) IκB-α, and IKK. The data shown represent mean ± S.D. of three independent experiments. ^#^ *p* < 0.05, ^###^ *p* < 0.001 vs. the control group; ** *p* < 0.01, *** *p* < 0.001 vs. TNF-α/IFN-γ-stimulated group.

It is known that epidermal keratinocytes have various roles in the immune diseases and are associated with AD and other skin diseases [20]. Therefore, we investigated the effect of HT on the human skin keratinocyte in vitro. The optimal treatment concentration of HT on HaCaT keratinocytes was determined by using MTT assay. HT had no cytotoxic effect on HaCaT keratinocytes at concentrations of 7.8 to 500 μg/mL after 24 h of treatment (Figure 5A). Thus, we used HT at concentrations of 100, 200, and 400 μg/mL for subsequent experiments. The protein expression of filaggrin was assessed using western blot analysis. HT was able to rescue filaggrin expression in TNF-α/IFN-γ-stimulated HaCaT keratinocytes. In the dermis, an upregulation of ICAM-1 expression occurs in endothelial cells activated by cytokines, such as IL-1 or TNF-α, and is usually correlated with the induction of VCAM-1. Pretreatment with 100, and 400 μg/mL of HT decreased VCAM-1 expression in TNF-α/IFN-γ-stimulated HaCaT keratinocytes (Figure 5B). However, significant increases in the levels of ICAM-1 and E-selectin were not observed in TNF-α/IFN-γ-stimulated HaCaT keratinocytes. These in vitro results supported that filaggrin and adhesion molecules are involved in anti-AD mechanisms of HT.

### 3.6. HT Suppressed the Activation of the NF-κB Signaling Pathway in TNF-α/IFN-γ-Stimulated HaCaT Keratinocytes

Once activated, NF-κB mediates the expression of various pro-inflammatory mediators during the inflammatory process. We next explored the effect of HT on the NF-κB signaling pathway in TNF-α/IFN-γ-stimulated HaCaT keratinocytes using western blot analysis. Basal levels of p65 were detected in the nuclear protein of unstimulated cells, but treatment of cells with TNF-α/IFN-γ induced the nuclear translocation of p65. Pre-treatment of keratinocytes with 200, and 400 μg/mL of HT reduced nuclear translocation of NF-κB while 100 μg/mL of HT inhibited this event in TNF-α/IFN-γ-stimulated HaCaT keratinocytes (Figure 5C). It is well documented that IKK and IκBα signaling pathways mediate NF-κB signaling in TNF-α/IFN-γ-stimulated HaCaT keratinocyte [21]. Since nuclear translocation of NF-κB is preceded by phosphorylation/degradation of IκBα, and IKK activates IκBα, we investigated IκBα and IKK phosphorylation to elucidate the inhibitory effect of HT in the upstream event of NF-κB. Pre-treatment of keratinocytes with HT also attenuated TNF-α/IFN-γ-stimulated IκBα and IKK phosphorylation (Figure 5D). These results indicate that the expression levels of filaggrin and adhesion molecules are related to the NF-κB signaling pathway.

### 3.7. HT Suppressed the Phosphorylation of the Akt and MAPK Signaling Pathways in TNF-α/IFN-γ-Stimulated HaCaT Keratinocytes

**Figure 6:**
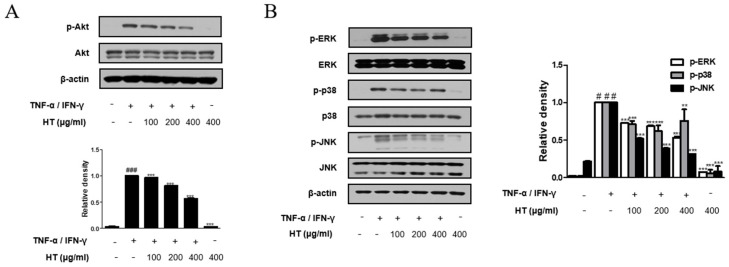
Effect of HT on the phosphorylation Akt and MAPK in TNF-α/IFN-γ-stimulated HaCaT keratinocytes. HaCaT keratinocytes were pre-treated with HT for 1 h and then stimulated with TNF-α/IFN-γ (10 ng/mL) for 5 min. Total proteins were prepared and western blotted for (**A**) Akt and (**B**) MAPK family using specific antibodies. β-actin was used as internal control. The data shown represent mean ± S.D. of three independent experiments. ^#^ *p* < 0.05, ^###^ *p* < 0.001 vs. the control group; ** *p* < 0.01, and *** *p* < 0.001 vs. TNF-α/IFN-γ-stimulated group.

Akt is involved in promoting growth arrest, proliferation, and differentiation in keratinocytes [22]. Therefore, we investigated the Akt signal pathway, another signaling component upstream of the NF-κB signaling pathway, using western blot analysis. As shown in Figure 6A, HT suppressed TNF-α/IFN-γ-induced phosphorylation of Akt at Ser473 in a dose-dependent manner. We also investigated whether HT affected the expression of the MAPKs, which are important mediators of AD, since phosphorylation of MAPKs induces cytokine production [23]. HT decreased TNF-α/IFN-γ-induced phosphorylation of ERK, p38, and JNK (Figure 6B). Together, these results suggest that the regulation of TNF-α/IFN-γ-induced decreases in filaggrin and increases in the expression of adhesion molecules by HT is mediated by the suppression of both Akt and MAPK activation.

## 4. Discussion

AD is the most common inflammatory skin disease with a broad spectrum of clinical skin phenotypes. The pathology of AD skin is characterized by epidermal intercellular edema and barrier dysfunction resulting in increased transepidermal water loss, marked mononuclear cell infiltration in the dermis, and increased penetration of external allergens through the skin barrier [24]. In our study, topical application of HT diminished the symptoms of AD in the Dfb-induced AD model, including epidermal thickness, mast cell infiltration, pro-inflammatory cytokine production, and serum histamine and IgE levels. In the Dfb-induced AD model, treatment with HT had a similar or better anti-AD effect than dexamethasone. However, HT had a weak effect on the reduction in size and weight of the spleen and lymph nodes, which may be involved in immune activity. This result is probably related to immune activation involving various immune cells in the development of AD (Figure 1 and Figure 3).

The enzyme allergens in house dust mite extracts may enhance the penetration of allergens through the altered skin epidermis and are known to be inducers of the production of multiple cytokines, including interleukin (IL)-6, IL-8, and granulocyte/macrophage colony-stimulating factor (GM-CSF) [25]. Keratinocytes are the source of cytokines including inflammatory cytokines (IL-6, IL-1α, IL-1β, IL-18, and TNF-α), immunoregulatory cytokines (IL-7, IL-12, IL-15, IL-16, and GM-CSF), and chemokines (IL-8, TARC, RANTES, and MCP-1) in AD skin. Among these cytokines, the role of IL-6 is critical in AD skin [26]. Moreover, IL-6 activates Th2 cells, thus leading to secretion of IL-4 and IL-13 and promoting a Th2 immune response. This is followed by stimulating B cells to produce IgE that binds to mast cells [27]. As shown in Figure 2, we observed that topically administration of HT markedly decresed Dfb-induced IL-6, histamine, and IgE levels in Dfb-induced AD mice, but had a little effect on the expression of TNF-α. It is estimated that this result comes from the complex mechanism of skin in which various cells interact. Among these mechanisms of skin, HT has shown a particularly inhibiting effect on the IL-6, histamine, and IgE levels that can lead to the development of AD symptoms in Dfb-induced AD mice.

AD primarily occurs through defects in the immune system, leading to excessive inflammation and local epidermal barrier disruption [28]. Filaggrin is expressed originally as a profilaggrin, which is cleaved to produce monomers that align to form keratin filaments. Filaggrin is degraded in a multistep proteolysis to release hygroscopic amino acids, contributing to the moisture in the skin [29]. It is reported that upregulation of inflammatory cytokines may play an important role in the downregulation of filaggrin in keratinocytes [30]. Moreover, recent studies indicate that filaggrin expression can be modulated by the atopic inflammatory response mediated by inflammatory cytokines, thus providing a link between these structural elements and the inflammatory response in AD skin [31]. For example, IL-6 and IL-6 receptor expression is localized to all nucleated epidermal layers in case of barrier disruption. IL-6 is able to increase keratinocyte proliferation and epidermal thickening in a transgenic mouse model [32]. This can be explained, at least in part, by the finding that IL-6 promotes Dfb-induced AD skin symptoms. It is also reported that increased IL-6 levels could induce the disruption of the skin barrier in AD patients and the concomitant enhanced risk of pathogen invasion that promotes the production of IL-6 by immune cells [33]. Our finding is consistent with previously reported findings suggesting an inverse relationship between IL-6 and filaggrin expression [34,35].

Cell adhesion molecules play a key role in several pathologies, such as cancer and inflammatory diseases [36]. It has long been known that pathological cell adhesion is observed between nerve fibers and mast cells in AD skin lesions [37]. ICAM-1, VCAM-1, and selectins are closely related to allergic skin diseases and their levels are consequentially increased in the epidermis of patients with AD; Th2 cytokines are recruited to the inflamed site by these inflammatory factors [38]. In the present study, we found that HT treatment reduced the protein levels of adhesion molecules in Dfb-induced AD mice model (Figure 4). Moreover, the expression of multifold adhesion molecules on human keratinocytes is induced by pro-inflammatory cytokines such as TNF-α and IFN-γ [39], wheareas HT suppressed the increased protein expression of VCAM-1 (Figure 5B). Unexpectedly, ICAM-1 and E-selectin were not detected in TNF-α and IFN-γ-stimulated HaCaT keratinocytes. It is likely that the complex environment of the skin tissue involving various cell types (e.g., fibroblasts, leukocytes) rather a monoculture would have a greater impact on the expression of the adhesion molecules.

We also investigated the effect of HT and its underlying mechanisms in TNF-α/IFN-γ-stimulated human keratinocyte. We showed that the reduction in expression of degraded filaggrin and increase in adhesion molecules were reversed by HT pre-treatment in TNF-α/IFN-γ-stimulated HaCaT keratinocytes. It is reported the expression of adhesion molecules is regulated by the intracellular signaling pathways, including MAPK and protein kinase C pathways, involving the transcription factors NF-κB, activator protein 1, signal transducer and activator of transcription, and CCAAT-enhancer-binding proteins. The signaling pathways activated depend on the stimuli, or agonist, and may differ between cell types [40,41,42]. In this study, HT suppressed the activation of NF-κB (Figure 5C,D) and phosphorylation of Akt and MAPK (Figure 6) signaling pathways in TNF-α/IFN-γ-stimulated HaCaT keratinocytes. The results suggest that the NF-κB, Akt, and MAPK signaling pathways participate in the inhibition of the expression of filaggrin and adhesion molecules by HT.

In the present study, we proposed several mechanisms by which HT inhibits AD skin symptoms during the allergic inflammatory response. Together with adhesion molecules, pro-inflammatory cytokines/chemokines, such as IL-6, MCP-1, and IP-10, play important roles in immune cell infiltration of the inflamed site in the skin during immune responses [21]. Dysregulation of chemokines, cytokines, and adhesion molecules generally enhance these conditions, resulting in the development of AD [43]. Previous studies also have revealed that elevated plasma concentrations of adhesion molecules are related to IL-6 in patients with various diseases, including carotid atherosclerosis, coronary artery disease [44], and migraine [45]. Therefore, the behavior of cytokines and soluble inflammatory mediators, the association between the levels of the inflammatory factors, such as IL-6, filaggrin, and cell adhesion molecules, and the mechanisms underlying allergic inflammatory diseases require further investigation. In addition, one recent study has suggested a role for the anti-oxidative axis in skin barrier dysfunction [46,47]. In this regard, the effect of HT on oxidative stress related to filaggrin and the other differentiation markers of keratinocytes in the AD model should also be further investigated to better understand the effect of HT on AD.

## 5. Conclusions

**Figure 7:**
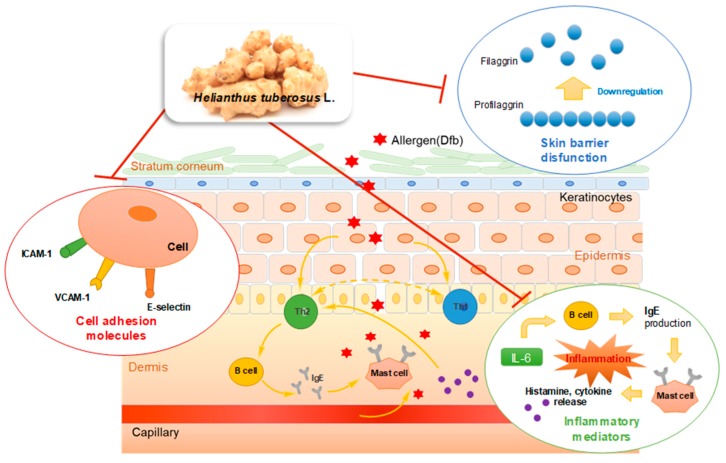
Graphical Abstract: Underlying molecular mechanisms in the anti-AD effect of HT.

We demonstrated that topical application of HT effectively attenuated Dfb-induced AD skin symptoms by restoring skin barrier functions and inhibiting Dfb-induced increases in inflammatory mediators, adhesion molecules, and Dfb-induced histopathological changes. HT treatment not only regulated the inflammatory cytokines and adhesion molecules, which are implicated in the inflammatory response, but also enhanced the expression of the skin barrier protein, filaggrin. These effects of HT were also confirmed by an in vitro study using human skin keratinocytes. We described the results of this study in Figure 7. The results provide evidence that supports the therapeutic use of HT for the clinical treatment of AD and related inflammatory skin diseases. Based on these results, we conclude that HT has promising potential as a novel agent or food supplement for the prevention and treatment of AD.

## References

[1] Jin M., Choi J.K., Choi Y.A., Kim Y.Y., Baek M.C., Lee B.H., Jang Y.H., Lee W.J., Lee S.J., Kim do W. (2016). 1,2,4,5-Tetramethoxybenzene suppresses house dust mite-induced allergic inflammation in BALB/c mice. Int. Arch. Allergy Immunol..

[2] Choi J.K., Oh H.M., Lee S., Kwon T.K., Shin T.Y., Rho M.C., Kim S.H. (2014). Salvia plebeia suppresses atopic dermatitis-like skin lesions. Am. J. Chin. Med..

[3] Gao X.K., Nakamura N., Fuseda K., Tanaka H., Inagaki N., Nagai H. (2004). Establishment of allergic dermatitis in NC/Nga mice as a model for severe atopic dermatitis. Biol. Pharm Bull..

[4] Cabanillas B., Novak N. (2016). Atopic dermatitis and filaggrin. Curr. Opin. Immunol..

[5] Lee C.W., Lin Z.C., Hu S.C., Chiang Y.C., Hsu L.F., Lin Y.C., Lee I.T., Tsai M.H., Fang J.Y. (2016). Urban particulate matter down-regulates filaggrin via COX2 expression/PGE2 production leading to skin barrier dysfunction. Sci. Rep..

[6] Lugovic L., Cupic H., Lipozencic J., Jakic-Razumovic J. (2006). The role of adhesion molecules in atopic dermatitis. Acta Dermatovenerol. Croat..

[7] Jung K., Linse F., Heller R., Moths C., Goebel R., Neumann C. (1996). Adhesion molecules in atopic dermatitis: VCAM-1 and ICAM-1 expression is increased in healthy-appearing skin. Allergy.

[8] Chang W.C., Jia H., Aw W., Saito K., Hasegawa S., Kato H. (2014). Beneficial effects of soluble dietary Jerusalem artichoke (Helianthus tuberosus) in the prevention of the onset of type 2 diabetes and non-alcoholic fatty liver disease in high-fructose diet-fed rats. Br. J. Nutr..

[9] Radulovic N.S., Dordevic M.R. (2014). Chemical composition of the tuber essential oil from Helianthus tuberosus L. (Asteraceae). Chem. Biodivers..

[10] Chen F., Long X., Liu Z., Shao H., Liu L. (2014). Analysis of phenolic acids of Jerusalem artichoke (Helianthus tuberosus L.) responding to salt-stress by liquid chromatography/tandem mass spectrometry. Sci. World J..

[11] Wang Y.M., Zhao J.Q., Yang J.L., Idong P.T., Mei L.J., Tao Y.D., Shi Y.P. (2017). Antioxidant and alpha-glucosidase inhibitory ingredients identified from Jerusalem artichoke flowers. Nat. Prod. Res..

[12] Yang L., He Q.S., Corscadden K., Udenigwe C.C. (2015). The prospects of Jerusalem artichoke in functional food ingredients and bioenergy production. Biotechnol. Rep. (Amst.).

[13] Yang G., Cheon S.Y., Chung K.S., Lee S.J., Hong C.H., Lee K.T., Jang D.S., Jeong J.C., Kwon O.K., Nam J.H. (2015). *Solanum tuberosum* L. cv jayoung epidermis extract inhibits mite antigen-induced atopic dermatitis in NC/Nga mice by regulating the Th1/Th2 balance and expression of filaggrin. J. Med. Food.

[14] Lee H.S., Choi E.J., Lee K.S., Kim H.R., Na B.R., Kwon M.S., Jeong G.S., Choi H.G., Choi E.Y., Jun C.D. (2016). Oral administration of p-hydroxycinnamic acid attenuates atopic dermatitis by downregulating Th1 and Th2 cytokine production and keratinocyte activation. PLoS ONE.

[15] Arts J.H., Droge S.C., Bloksma N., Kuper C.F. (1996). Local lymph node activation in rats after dermal application of the sensitizers 2,4-dinitrochlorobenzene and trimellitic anhydride. Food Chem. Toxicol..

[16] Purushothaman B., Arumugam P., Song J.M. (2018). A novel catecholopyrimidine based small molecule PDE4B inhibitor suppresses inflammatory cytokines in atopic mice. Front. Pharmacol..

[17] Limandjaja G.C., van den Broek L.J., Waaijman T., van Veen H.A., Everts V., Monstrey S., Scheper R.J., Niessen F.B., Gibbs S. (2017). Increased epidermal thickness and abnormal epidermal differentiation in keloid scars. Br. J. Dermatol..

[18] Irvine A.D., McLean W.H., Leung D.Y. (2011). Filaggrin mutations associated with skin and allergic diseases. N. Engl. J. Med..

[19] Yang I.J., Lee D.U., Shin H.M. (2016). Inhibitory effect of valencene on the development of atopic dermatitis-like skin lesions in NC/Nga mice. Evid. Based Complement. Altern. Med..

[20] Lee J.H., Jeon Y.D., Lee Y.M., Kim D.K. (2018). The suppressive effect of puerarin on atopic dermatitis-like skin lesions through regulation of inflammatory mediators in vitro and in vivo. Biochem. Biophys. Res. Commun..

[21] Seo W.Y., Youn G.S., Choi S.Y., Park J. (2015). Butein, a tetrahydroxychalcone, suppresses pro-inflammatory responses in HaCaT keratinocytes. BMB Rep..

[22] Segrelles C., Ruiz S., Santos M., Martinez-Palacio J., Lara M.F., Paramio J.M. (2004). Akt mediates an angiogenic switch in transformed keratinocytes. Carcinogenesis.

[23] Lim J.M., Lee B., Min J.H., Kim E.Y., Kim J.H., Hong S., Kim J.J., Sohn Y., Jung H.S. (2018). Effect of peiminine on DNCB-induced atopic dermatitis by inhibiting inflammatory cytokine expression in vivo and in vitro. Int. Immunopharmacol..

[24] Cole C., Kroboth K., Schurch N.J., Sandilands A., Sherstnev A., O’Regan G.M., Watson R.M., McLean W.H., Barton G.J., Irvine A.D. (2014). Filaggrin-stratified transcriptomic analysis of pediatric skin identifies mechanistic pathways in patients with atopic dermatitis. J. Allergy Clin. Immunol..

[25] Arlian L.G., Morgan M.S., Peterson K.T. (2008). House dust and storage mite extracts influence skin keratinocyte and fibroblast function. Int. Arch. Allergy Immunol..

[26] Yamanaka K., Mizutani H. (2011). The role of cytokines/chemokines in the pathogenesis of atopic dermatitis. Curr. Probl. Dermatol..

[27] Kwon T.R., Mun S.K., Oh C.T., Hong H., Choi Y.S., Kim B.J., Kim B.J. (2014). Therapeutic effects of full spectrum light on the development of atopic dermatitis-like lesions in NC/Nga mice. Photochem. Photobiol..

[28] Pendaries V., Malaisse J., Pellerin L., Le Lamer M., Nachat R., Kezic S., Schmitt A.M., Paul C., Poumay Y., Serre G. (2014). Knockdown of filaggrin in a three-dimensional reconstructed human epidermis impairs keratinocyte differentiation. J. Investig. Dermatol..

[29] O’Regan G.M., Sandilands A., McLean W.H., Irvine A.D. (2008). Filaggrin in atopic dermatitis. J. Allergy Clin. Immunol..

[30] Jin S.H., Choi D., Chun Y.J., Noh M. (2014). Keratinocyte-derived IL-24 plays a role in the positive feedback regulation of epidermal inflammation in response to environmental and endogenous toxic stressors. Toxicol. Appl. Pharmacol..

[31] Howell M.D., Kim B.E., Gao P., Grant A.V., Boguniewicz M., DeBenedetto A., Schneider L., Beck L.A., Barnes K.C., Leung D.Y. (2009). Cytokine modulation of atopic dermatitis filaggrin skin expression. J. Allergy Clin. Immunol..

[32] Sawamura D., Meng X., Ina S., Sato M., Tamai K., Hanada K., Hashimoto I. (1998). Induction of keratinocyte proliferation and lymphocytic infiltration by in vivo introduction of the IL-6 gene into keratinocytes and possibility of keratinocyte gene therapy for inflammatory skin diseases using IL-6 mutant genes. J. Immunol..

[33] Hanel K.H., Cornelissen C., Luscher B., Baron J.M. (2013). Cytokines and the skin barrier. Int. J. Mol. Sci..

[34] Noh M., Yeo H., Ko J., Kim H.K., Lee C.H. (2010). MAP17 is associated with the T-helper cell cytokine-induced down-regulation of filaggrin transcription in human keratinocytes. Exp. Dermatol..

[35] Son E.D., Kim H.J., Park T., Shin K., Bae I.H., Lim K.M., Cho E.G., Lee T.R. (2014). Staphylococcus aureus inhibits terminal differentiation of normal human keratinocytes by stimulating interleukin-6 secretion. J. Dermatol. Sci..

[36] Golias C., Tsoutsi E., Matziridis A., Makridis P., Batistatou A., Charalabopoulos K. (2007). Review. Leukocyte and endothelial cell adhesion molecules in inflammation focusing on inflammatory heart disease. In Vivo.

[37] Yoneshige A., Hagiyama M., Fujita M., Ito A. (2015). Pathogenic actions of cell adhesion molecule 1 in pulmonary emphysema and atopic dermatitis. Front. Cell. Dev. Biol..

[38] Gao C.J., Ding P.J., Yang L.L., He X.F., Chen M.J., Wang D.M., Tian Y.X., Zhang H.M. (2018). Oxymatrine sensitizes the HaCaT cells to the IFN-gamma pathway and downregulates MDC, ICAM-1, and SOCS1 by activating p38, JNK, and Akt. Inflammation.

[39] Sung Y.Y., Yoon T., Jang S., Kim H.K. (2016). Forsythia suspensa suppresses house dust mite extract-induced atopic dermatitis in NC/Nga mice. PLoS ONE.

[40] Waters C.E., Shi-Wen X., Denton C.P., Abraham D.J., Pearson J.D. (2006). Signaling pathways regulating intercellular adhesion molecule 1 expression by endothelin 1: Comparison with interleukin-1beta in normal and scleroderma dermal fibroblasts. Arthritis Rheum..

[41] Liu Y., Kimura K., Yanai R., Chikama T., Nishida T. (2008). Cytokine, chemokine, and adhesion molecule expression mediated by MAPKs in human corneal fibroblasts exposed to poly(I:C). Investig. Ophthalmol. Vis. Sci..

[42] Shi Y., Xia Y.Y., Wang L., Liu R., Khoo K.S., Feng Z.W. (2012). Neural cell adhesion molecule modulates mesenchymal stromal cell migration via activation of MAPK/ERK signaling. Exp. Cell. Res..

[43] Homey B., Steinhoff M., Ruzicka T., Leung D.Y. (2006). Cytokines and chemokines orchestrate atopic skin inflammation. J. Allergy Clin. Immunol..

[44] Signorelli S.S., Mazzarino M.C., Di Pino L., Malaponte G., Porto C., Pennisi G., Marchese G., Costa M.P., Digrandi D., Celotta G. (2003). High circulating levels of cytokines (IL-6 and TNFalpha), adhesion molecules (VCAM-1 and ICAM-1) and selectins in patients with peripheral arterial disease at rest and after a treadmill test. Vasc. Med..

[45] Wang F., He Q., Ren Z., Li F., Chen W., Lin X., Zhang H., Tai G. (2015). Association of serum levels of intercellular adhesion molecule-1 and interleukin-6 with migraine. Neurol. Sci..

[46] Ji H., Li X.K. (2016). Oxidative stress in atopic dermatitis. Oxid. Med. Cell. Longev..

[47] van den Bogaard E.H., Bergboer J.G., Vonk-Bergers M., van Vlijmen-Willems I.M., Hato S.V., van der Valk P.G., Schroder J.M., Joosten I., Zeeuwen P.L., Schalkwijk J. (2013). Coal tar induces AHR-dependent skin barrier repair in atopic dermatitis. J. Clin. Investig..

